# THE LONGEVITY CONSORTIUM: MULTI-OMICS INTEGRATIVE APPROACH TO DISCOVERING HEALTHY AGING AND LONGEVITY DETERMINANTS

**DOI:** 10.1093/geroni/igz038.757

**Published:** 2019-11-08

**Authors:** Thomas T Perls, Daniel S Evans, Evan Hadley

**Affiliations:** 1 Boston University School of Medicine, Boston, Massachusetts, United States; 2 California Pacific Medical Center Research Institute, San Francisco, California, United States; 3 National Institute on Aging, National Institutes of Health, Bethesda, Maryland, United States

## Abstract

The Longevity Consortium (LC), a NIA-Cooperative project, is an integrated multi-disciplinary effort with cutting edge bioinformatic, systems biology and chemoinformatics approaches exploiting multiple omics data generated from multiple well-phenotyped aging cohorts, Study of Osteoporotic Fractures, MrOS (fractures in men), Cardiovascular Health Study, the Long Life Family Study and the Centenarian Project to discover pharmacologically targetable protective pathways that promote healthy aging. Omics studies of mice treated with candidate drugs and of multiple species with varying life spans further informs the LC efforts. We describe the integration of the above efforts and the LC goals as well as opportunities for interested investigators to access shared results as well as opportunity funds for pilot projects. Early successes are described in 4 presentations: (1) A genome-wide association study including 1317 centenarians discovered 8 new loci in chromosomes 3, 6, 7, 9, 10, 14 and 15. The list includes new serum pQTLs that suggest a new biological mechanism involved in extreme longevity. (2) Novel, high-throughput discovery-proteomics of serum from 2,473 MrOs participants identified 25 proteins, mostly found in inflammatory pathways, associated with living beyond the 90th percentile birth-cohort survival. (3) A biological age estimation algorithm utilizing multi-omics assays significantly differentiated between 3,558 wellness program participants and controls. (4) Mediation analyses were used to test causal relationships between many candidate aging-modulating drugs and compounds, expression levels of gene and protein variants (eQTLs and pQTLs) and aging and longevity phenotypes. This high throughput method shows promise as a means of discovering candidate drugs for healthy aging.

